# Supplementary material for: Characteristics and Treatment of Primary Hepatic Perivascular Epithelioid Cell Tumor (PEComa) in Adults: A Systematic Review
Source: Cancers (Basel). 2025 Jul 8;17(14):2276. doi: 10.3390/cancers17142276 (PMC12294052; doi:10.3390/cancers17142276)
Supplement: Supplementary file 1 [file cancers-17-02276-s001.zip › cancers-3713775-supplementary.pdf]

Supplementary Materials: Characteristics and Treatment of Primary Hepatic Perivascular Epithelioid Cell Tumor (PEComa) in Adults: A Systematic Review

Konstantinos Papantoniou, Ioanna Aggeletopoulou, Maria Kalafateli and Christos Triantos

Table S1. Characteristics of patients included in the analysis. References [11-155] are included in this table.

Author	Year	Continent	Sex	Lobe	Size (cm)	PEComa type	Method of diagnosis	Malignancy	Surgery	FU (months)
Kudo	1993	Asia	F	R	1,3	AML	Post-surgical analysis	No	Yes	18
Yamada	1993	Asia	M	R	0,9	AML	Biopsy	No	No	10
Carmody	1994	North America	F	R,L	6	AML	Radiology	No	No	48
Carmody	1993	North America	M	R,L	3	AML	Radiology	No	No	36
Kimura	1994	Asia	F	R,L	4	AML	Post-surgical analysis	No	Yes	6
Peh	1995	Asia	F	R	9	AML	Post-surgical analysis	No	Yes	5
Peh	1995	Asia	F	R,L	14	AML	Biopsy	No	Yes	6
Hoffmann	1997	North America	F	C	4	AML	Post-surgical analysis	No	Yes	30
Hoffmann	1997	North America	F	C	4,5	AML	Post-surgical analysis	No	Yes	68
Sawai	1998	Asia	M	L	4	AML	Biopsy	Yes	Yes	10
Rocken	1999	Europe	F	L	13	AML	Post-surgical analysis	No	Yes	28
Sajima	1999	Asia	M	R	3,5	AML	Post-surgical analysis	No	Yes	20
Dalle	2000	Europe	F	R	26	AML	Biopsy	Yes	Yes	13
Yamasaki	2000	Asia	F	R	3,2	AML	Post-surgical analysis	No	Yes	12
Ji	2001	Asia	M	R	4	AML	Post-surgical analysis	No	Yes	40
Ji	2001	Asia	F	R	5	AML	Post-surgical analysis	No	Yes	17
Ji	2001	Asia	F	L	2	AML	Post-surgical analysis	No	Yes	12
Ji	2001	Asia	F	L	4	AML	Post-surgical analysis	No	Yes	14
Ji	2001	Asia	F	R	1,5	AML	Post-surgical analysis	No	Yes	9
Ji	2001	Asia	F	R	2	AML	Post-surgical analysis	No	Yes	8
Ji	2001	Asia	M	R	7	AML	Post-surgical analysis	No	Yes	5
Ji	2001	Asia	M	R	8	AML	Post-surgical	No	Yes	4

							analysis			
Ji	2001	Asia	F	R	3	AML	Post-surgical analysis	No	Yes	2
Tang	2002	North America	F	R	20	AML	Post-surgical analysis	No	Yes	48
Rimola	2003	Europe	F	R	5	AML	Post-surgical analysis	No	No	96
Akatsu	2004	Asia	F	L	10	AML	Biopsy	No	Yes	48
Chao	2004	Asia	F	R	10	AML	Post-surgical analysis	No	No	12
De Bruecker	2004	Europe	F	L	4	AML	Post-surgical analysis	No	Yes	6
Kojima	2004	Asia	F	L	7	AML	Post-surgical analysis	No	Yes	65
Lin	2004	Asia	F	L	4,4	AML	Biopsy	No	No	8
Lin	2004	Asia	M	R	3	AML	Biopsy	No	No	21
Romano	2004	Europe	F	L	12	AML	Biopsy	No	Yes	12
Saito	2004	Asia	F	L	6	AML	Post-surgical analysis	No	Yes	96
Sebastian	2004	Europe	F	R	7	AML	Biopsy	No	No	5
Tryggvason	2004	Europe	F	L	7,2	AML	Post-surgical analysis	No	Yes	23
Takamura	2005	Asia	M	R	4	AML	Biopsy	No	No	84
Yen	2005	Asia	M	R	2,7	AML	Biopsy	No	No	24
Flor	2006	Europe	F	R	6,4	AML	Post-surgical analysis	No	Yes	22
Parfitt	2006	Europe	F	R	14	AML	Post-surgical analysis	Yes	Yes	120
Wang	2006	Asia	M	L	5,4	AML	Post-surgical analysis	No	Yes	38
Fang	2007	Asia	F	L	5,1	PEComa-NOS	Post-surgical analysis	No	Yes	24
Fang	2007	Asia	F	C	999	PEComa-NOS	Post-surgical analysis	No	No	12
Larbcharoensub	2007	Asia	F	R	18	CCMMT of falciform ligament	Post-surgical analysis	No	Yes	6
Yang	2007	Asia	F	R	10	AML	Biopsy	No	Yes	39
Yang	2007	Asia	F	R	13	AML	Radiology	No	Yes	59
Yang	2007	Asia	F	L	20	AML	Radiology	Yes	Yes	14
Yang	2007	Asia	F	R	7	AML	Post-surgical analysis	No	Yes	109
Yang	2007	Asia	F	R	11	AML	Biopsy	No	No	6
Yang	2007	Asia	F	L	15	AML	Post-surgical analysis	No	Yes	40
Yang	2007	Asia	F	R	3	AML	Post-surgical analysis	No	Yes	37
Yang	2007	Asia	F	C	2,5	AML	Post-surgical analysis	No	Yes	40
Yang	2007	Asia	F	L	4	AML	Post-surgical analysis	No	Yes	33
Yang	2007	Asia	M	L		AML	Post-surgical analysis	No	Yes	32
Della Vigna	2008	Europe	F	L	3,5	PEComa-NOS	Biopsy	No	Yes	5

Deng	2008	Asia	M	R	18	AML	Radiology	Yes	Yes	40
Lenci	2008	Europe	F	R	12	AML	Biopsy	No	No	12
Nguyen	2008	North America	F	L	11	AML	Post-surgical analysis	Yes	Yes	10
Paiva	2008	South America	F	L	0,8	PEComa-NOS	Post-surgical analysis	No	No	25
Yang	2008	Asia	F	R,L	.	AML	Radiology	No	No	12
Zimmermann	2008	Europe	M	R	7	PEComa-NOS	Radiology	No	No	17
Akitake	2009	Asia	F	L	3,5	PEComa-NOS	Radiology	No	Yes	18
Chen	2009	Asia	F	R	3	AML	Post-surgical analysis	No	Yes	6
Priola	2009	Europe	F	L	11	CCMMT of falciform ligament	Post-surgical analysis	No	Yes	34
Strzelczyk	2009	Europe	F	R	17	clear cell sugar tumor	Post-surgical analysis	No	Yes	53
Wang	2009	Asia	F	N/A	3	AML	Biopsy	No	No	24
Wang	2009	Asia	F	L	2,7	AML	Biopsy	No	No	30
Kamimura	2009	Asia	M	L	2	AML	Post-surgical analysis	Yes	Yes	36
Shi	2010	Asia	F	L	5,5	AML	Post-surgical analysis	No	Yes	60
Shi	2010	Asia	F	R	7,5	AML	Post-surgical analysis	No	Yes	84
Shi	2010	Asia	M	L	8	AML	Post-surgical analysis	No	Yes	48
Shi	2010	Asia	F	L	6,3	AML	Post-surgical analysis	No	Yes	36
Shi	2010	Asia	F	R	10	AML	Post-surgical analysis	No	Yes	108
Zeng	2010	Asia	M	L	9	AML	Post-surgical analysis	No	Yes	29
Zeng	2010	Asia	M	L	3,5	AML	Post-surgical analysis	No	Yes	31
Zeng	2010	Asia	M	L	6	AML	Post-surgical analysis	No	Yes	141
Zeng	2010	Asia	M	L	3	AML	Post-surgical analysis	No	Yes	44
Zeng	2010	Asia	M	L	2,5	AML	Post-surgical analysis	No	Yes	29
Zeng	2010	Asia	M	L	22	AML	Radiology	No	Yes	82
Zeng	2010	Asia	F	L	6	AML	Post-surgical analysis	No	Yes	86
Zeng	2010	Asia	F	L	3,5	AML	Post-surgical analysis	No	Yes	26
Zeng	2010	Asia	F	R	11	AML	Biopsy	No	Yes	60
Zeng	2010	Asia	F	R	4	AML	Biopsy	No	Yes	79
Zeng	2010	Asia	F	R	14	AML	Post-surgical analysis	No	Yes	115
Zeng	2010	Asia	F	R	19	AML	Post-surgical analysis	No	Yes	106
Zeng	2010	Asia	F	R	2	AML	Post-surgical analysis	No	Yes	58
Zeng	2010	Asia	F	R	14	AML	Post-surgical	No	Yes	58

							analysis			
Zeng	2010	Asia	F	R	8	AML	Post-surgical analysis	No	Yes	54
Zeng	2010	Asia	F	C	17	AML	Post-surgical analysis	No	Yes	117
Zeng	2010	Asia	F	C	12,5	AML	Post-surgical analysis	No	Yes	120
Ahn	2011	Asia	F	L	6,5	AML	Post-surgical analysis	No	Yes	3
Mima	2011	Asia	F	L	2	AML	Post-surgical analysis	No	Yes	29
Selvaggi	2011	Europe	M	R	7	PEComa-NOS	Post-surgical analysis	Yes	Yes	Death during hospitalization
Tani	2011	Asia	F	L	1,5	AML	Post-surgical analysis	No	Yes	12
Vagefi	2011	North America	M	L	20	AML	Biopsy	No	No	24
Agaimy	2012	Europe	M	R	7	AML	Post-surgical analysis	No	Yes	12
Agaimy	2012	Europe	M	L	18	AML	Post-surgical analysis	No	Yes	57
Agaimy	2012	Europe	F	L	2	AML	Post-surgical analysis	No	Yes	37
Agaimy	2012	Europe	M	L	1,1	AML	Biopsy	No	No	9
Costa	2012	Europe	F	L	6	AML	Radiology	No	Yes	36
Durczyński	2012	Europe	F	L	15	clear cell sugar tumor	Post-surgical analysis	No	Yes	65
Liu	2012	Asia	F	R	3	AML	Post-surgical analysis	No	Yes	24
Tan	2012	Asia	F	R	4	AML	Post-surgical analysis	No	Yes	48
Tan	2012	Asia	F	R	4,3	AML	Post-surgical analysis	Yes	Yes	14
Tan	2012	Asia	F	L	2,5	AML	Post-surgical analysis	No	Yes	32
Tan	2012	Asia	F	R	8	AML	Post-surgical analysis	No	Yes	36
Tan	2012	Asia	F	R	2,5	AML	Post-surgical analysis	No	Yes	27
Tan	2012	Asia	M	R	999	CCMMT of falciform ligament	Post-surgical analysis	No	Yes	19
Tan	2012	Asia	F	L	999	AML	Post-surgical analysis	Yes	Yes	12
Agaimy	2013	Europe	F	L	4,3	AML	Post-surgical analysis	No	Yes	84
Cheung	2013	Asia	F	R	10	PEComa-NOS	Post-surgical analysis	No	Yes	12
Jafari	2013	Europe	F	L	7	PEComa-NOS	Post-surgical analysis	No	Yes	14
Patra	2013	Asia	F	R,L	24	PEComa-NOS	Post-surgical analysis	No	Yes	24
Sheng	2013	Asia	M	R	2	PEComa-NOS	Post-surgical analysis	No	Yes	12
Shi	2013	Asia	F	R	12	PEComa-NOS	Post-surgical analysis	Yes	Yes	28

Shi	2013	Asia	F	R	10	PEComa-NOS	Post-surgical analysis	Yes	Yes	22
Shi	2013	Asia	F	R	15	PEComa-NOS	Post-surgical analysis	Yes	Yes	16
Shi	2013	Asia	M	C	13	PEComa-NOS	Post-surgical analysis	Yes	Yes	17
Tay	2013	Asia	F	L	9	PEComa-NOS	Post-surgical analysis	No	Yes	9
Yang	2013	Asia	F	R	5,3	AML	Post-surgical analysis	No	Yes	147
Yang	2013	Asia	F	R,L	4,5	AML	Biopsy	No	Yes	121
Yang	2013	Asia	M	L	8	AML	Post-surgical analysis	No	Yes	77
Yang	2013	Asia	M	R	3,2	AML	Post-surgical analysis	No	Yes	64
Yang	2013	Asia	F	L	4,7	AML	Post-surgical analysis	No	Yes	61
Yang	2013	Asia	F	R	1,5	AML	Post-surgical analysis	No	Yes	42
Yang	2013	Asia	F	L	2,3	AML	Post-surgical analysis	No	Yes	17
Yang	2013	Asia	F	L	2,6	AML	Biopsy	No	No	7
Yu	2013	Asia	F	R	1,9	PEComa-NOS	Post-surgical analysis	No	Yes	9
Zhao	2013	Asia	M	R	6	PEComa-NOS	Post-surgical analysis	No	Yes	9
Ameurtesse	2014	Africa	F	L	8	PEComa-NOS	Post-surgical analysis	No	Yes	9
Barbier	2014	Europe	F	R	11	AML	Post-surgical analysis	No	Yes	28
Kechaou	2014	Africa	F	R,L	9	AML	Radiology	No	No	48
Kumasaka	2014	Asia	M	R	1	AML	Radiology	No	Yes	48
Liu	2014	Asia	F	R	1,8	PEComa-NOS	Post-surgical analysis	No	Yes	12
Ortiz	2014	North America	F	L	7	CCMMT of falciform ligament	Post-surgical analysis	No	Yes	19
Sun	2014	Asia	F	L	5,5	AML	Post-surgical analysis	No	Yes	22
Tan	2014	Asia	F	R	8,5	CCMMT of falciform ligament	Post-surgical analysis	No	Yes	36
Wang	2014	Asia	F	R	10	AML	Post-surgical analysis	No	Yes	24
Wang	2014	Asia	M	L	15,3	AML	Radiology	No	Yes	12
Wang	2014	Asia	M	L	1,4	AML	Post-surgical analysis	No	Yes	12
Wang	2014	Asia	M	L	4	AML	Post-surgical analysis	No	Yes	10
Wang	2014	Asia	M	R,L	3,7	AML	Post-surgical analysis	No	Yes	10
Wang	2014	Asia	F	L	5,2	AML	Post-surgical analysis	No	Yes	2
Wang	2014	Asia	F	R	3,1	AML	Post-surgical analysis	No	Yes	10
Wang	2014	Asia	M	R	1,7	AML	Post-surgical	No	Yes	17

							analysis			
Wang	2014	Asia	F	L	6,5	AML	Post-surgical analysis	No	Yes	9
Yamaguchi	2014	Asia	M	L	7,5	AML	Post-surgical analysis	No	Yes	60
Zhou	2014	Asia	F	L	30	AML	Post-surgical analysis	No	Yes	71
Abhirup	2015	Asia	F	R	10	PEComa-NOS	Post-surgical analysis	Yes	Yes	12
Ge	2015	Asia	M	R	9	AML	Post-surgical analysis	No	Yes	24
Maebayashi	2015	Asia	M	L	4,5	PEComa-NOS	Post-surgical analysis	No	Yes	60
Neofytou	2015	Europe	F	R	1	AML	Post-surgical analysis	No	Yes	12
Wang	2015	Asia	F	L	9	AML	Post-surgical analysis	Yes	Yes	36
Zhou	2015	Asia	F	R	10	AML	Post-surgical analysis	No	Yes	11
Fukuda	2016	Asia	M	R	6,3	AML	Post-surgical analysis	Yes	Yes	108
Hao	2016	Asia	F	R	8	PEComa-NOS	Post-surgical analysis	No	Yes	6
Hao	2016	Asia	F	R	2,5	PEComa-NOS	Post-surgical analysis	No	Yes	8
Hao	2016	Asia	M	R	8	PEComa-NOS	Post-surgical analysis	No	Yes	36
Kirayama	2016	Asia	M	L	0,38	PEComa-NOS	Post-surgical analysis	No	Yes	56
Lan	2016	Asia	F	R,L	9,4	PEComa-NOS	Post-surgical analysis	No	Yes	12
Tang	2016	Asia	F	R	7	PEComa-NOS	Post-surgical analysis	No	Yes	9
Cardoso	2017	Europe	F	L	2,9	PEComa-NOS	Post-surgical analysis	No	Yes	36
Damascos	2017	Europe	F	L	4	AML	Post-surgical analysis	No	Yes	27
Guan	2017	Asia	F	R	7,5	PEComa-NOS	Biopsy	No	No	36
Hekimoglu	2017	Asia	F	R,C	5,2	PEComa-NOS	Biopsy	Yes	Yes	6
Kubo	2017	Asia	M	L	2,5	AML	Post-surgical analysis	No	No	12
Miyata	2017	Asia	M	L	15	AML	Radiology	No	Yes	24
Son	2017	Asia	F	R	4,5	PEComa-NOS	Post-surgical analysis	No	Yes	8
Jung	2018	Asia	M	R	17	AML	Radiology	No	Yes	99
Jung	2018	Asia	M	R	5,2	AML	Post-surgical analysis	No	Yes	94
Jung	2018	Asia	F	R	4,5	AML	Biopsy	No	Yes	81
Jung	2018	Asia	M	R	2,5	AML	Radiology	No	Yes	74
Jung	2018	Asia	F	R	18	AML	Radiology	No	Yes	73
Jung	2018	Asia	F	R	6,5	AML	Radiology	No	Yes	72
Jung	2018	Asia	F	R	2,3	AML	Post-surgical analysis	No	Yes	69
Jung	2018	Asia	F	R	7,5	AML	Post-surgical	No	Yes	65

							analysis			
Jung	2018	Asia	F	R	8,5	AML	Radiology	No	Yes	63
Jung	2018	Asia	F	R	8	AML	Biopsy	No	Yes	59
Jung	2018	Asia	F	L	3,5	AML	Post-surgical analysis	No	Yes	56
Jung	2018	Asia	F	R	0,9	AML	Post-surgical analysis	No	Yes	51
Jung	2018	Asia	F	L	2,7	AML	Biopsy	No	Yes	48
Jung	2018	Asia	F	R	1,1	AML	Post-surgical analysis	No	Yes	40
Jung	2018	Asia	M	R	3,5	AML	Post-surgical analysis	No	Yes	40
Jung	2018	Asia	M	L	8	AML	Post-surgical analysis	No	Yes	36
Jung	2018	Asia	F	R	4,3	AML	Biopsy	No	Yes	31
Jung	2018	Asia	F	R	6,4	AML	Biopsy	No	Yes	30
Jung	2018	Asia	F	R,L	3,6	AML	Post-surgical analysis	No	Yes	29
Jung	2018	Asia	M	R	0,6	AML	Post-surgical analysis	No	Yes	27
Jung	2018	Asia	F	R	2,7	AML	Radiology	No	Yes	23
Jung	2018	Asia	F	L	2,4	AML	Post-surgical analysis	No	Yes	21
Jung	2018	Asia	M	R	2,5	AML	Post-surgical analysis	No	Yes	19
Kirnap	2018	Asia	F	L	14	PEComa-NOS	Post-surgical analysis	Yes	Yes	10
Kirste	2018	Europe	F	R,L,C	N/A	PEComa-NOS	Biopsy	Yes	Yes	21
Ma	2018	Asia	F	R	15	AML	Radiology	No	Yes	12
Ma	2018	Asia	F	N/A	3	PEComa-NOS	Post-surgical analysis	No	Yes	12
Ma	2018	Asia	F	R	19,8	PEComa-NOS	Post-surgical analysis	No	Yes	18
Ma	2018	Asia	F	R	4	AML	Post-surgical analysis	No	Yes	60
Ma	2018	Asia	F	L	6	PEComa-NOS	Post-surgical analysis	No	Yes	18
Ma	2018	Asia	F	R	5	PEComa-NOS	Post-surgical analysis	No	Yes	3
Ma	2018	Asia	F	L	3,8	PEComa-NOS	Post-surgical analysis	No	Yes	15
Ma	2018	Asia	F	R,L	3,4	PEComa-NOS	Post-surgical analysis	No	Yes	2
Ma	2018	Asia	M	L	2	PEComa-NOS	Post-surgical analysis	No	Yes	2
Nell	2018	Australia	M	R	7	AML	Post-surgical analysis	No	Yes	24
Voulgari	2018	Europe	M	C	7	PEComa-NOS	Post-surgical analysis	No	Yes	18
Song	2019	Asia	F	R	8	PEComa-NOS	Post-surgical analysis	No	Yes	6
Britt	2020	North America	F	R	18,4	PEComa-NOS	Post-surgical analysis	Yes	Yes	192
Lopez	2020	Europe	F	L	1,5	PEComa-NOS	Post-surgical analysis	Yes	Yes	15

Lopez	2020	Europe	M	R	1,9	PEComa-NOS	Post-surgical analysis	Yes	Yes	11
Xu	2020	Asia	M	R	8	PEComa-NOS	Post-surgical analysis	No	Yes	14
Attard	2021	Europe	M	R	4,5	AML	Biopsy	No	Yes	20
Ergün	2021	Asia	F	L	12	CCMMT of falciform ligament	Post-surgical analysis	No	Yes	60
He	2021	Asia	F	R	2	PEComa-NOS	Biopsy	Yes	Yes	2
Huang	2021	Asia	M	C	3	AML	Post-surgical analysis	No	Yes	7
Li	2021	Asia	F	R	4,7	PEComa-NOS	Post-surgical analysis	No	Yes	16
Li	2022	Asia	F	R	18,9	PEComa-NOS	Biopsy	Yes	No	6
Wang	2022	Asia	F	L	5	AML	Post-surgical analysis	No	Yes	24
Zhang	2022	Asia	F	C	1	AML	Post-surgical analysis	No	Yes	6
Zhang	2022	Asia	F	R	1,2	AML	Post-surgical analysis	No	Yes	23
Zhu	2022	Asia	M	L	4	AML	Radiology	No	Yes	95,5
Zhu	2022	Asia	F	R	2,3	AML	Post-surgical analysis	No	Yes	67,5
Zhu	2022	Asia	F	L	8,3	AML	Post-surgical analysis	No	Yes	59,5
Zhu	2022	Asia	F	R	4,5	AML	Radiology	No	Yes	45,4
Zhu	2022	Asia	F	R	2,4	AML	Post-surgical analysis	No	Yes	27,9
Zhu	2022	Asia	F	R	3	AML	Post-surgical analysis	No	Yes	24,3
Zhu	2022	Asia	F	L,C	8	AML	Radiology	No	Yes	22,6
Zhu	2022	Asia	F	R	4	AML	Post-surgical analysis	No	Yes	22,1
Zhu	2022	Asia	F	L	13	AML	Post-surgical analysis	No	Yes	19,9
Zhu	2022	Asia	M	L	3,2	AML	Post-surgical analysis	No	Yes	11,2
Zhu	2022	Asia	F	R	7,5	AML	Radiology	No	Yes	9,4
Zhu	2022	Asia	F	L	16	AML	Radiology	No	Yes	5,6
Cai	2023	Asia	F	R	5,7	AML	Biopsy	Yes	No	12
Harwal	2023	Asia	F	R	13	PEComa-NOS	Post-surgical analysis	Yes	Yes	12
Ji	2023	Asia	F	C	20	AML	Post-surgical analysis	No	Yes	13
Kou	2023	Asia	M	R	2,5	PEComa-NOS	Post-surgical analysis	Yes	Yes	12
Kou	2023	Asia	F	L	5	PEComa-NOS	Post-surgical analysis	No	Yes	35
Kou	2023	Asia	F	R	7,8	PEComa-NOS	Post-surgical analysis	No	Yes	46
Li	2023	Asia	F	R	14,4	PEComa-NOS	Biopsy	Yes	No	6
Marinho	2023	Europe	F	R	2,3	AML	Post-surgical analysis	No	Yes	12

Matrood	2023	Europe	F	R	2	PEComa-NOS	Biopsy	No	No	24
Mochizuki	2023	Asia	F	R	1,2	AML	Post-surgical analysis	No	Yes	37
Wannasai	2023	Asia	F	R	4	PEComa-NOS	Post-surgical analysis	No	Yes	24
Yang	2023	Asia	M	L	2	PEComa-NOS	Post-surgical analysis	No	Yes	14
Costa	2024	North America	F	L	1,1	PEComa-NOS	Biopsy	No	Yes	9
Dymkowski	2024	Europe	M	R,L	2,7	PEComa-NOS	Post-surgical analysis	No	No	36
Jiang	2024	Asia	F	R	1,48	AML	Post-surgical analysis	No	Yes	38
Jiang	2024	Asia	F	R	1,5	AML	Post-surgical analysis	No	Yes	17
Jiang	2024	Asia	F	R	8,4	AML	Post-surgical analysis	No	Yes	40
Jiang	2024	Asia	M	L	14,1	AML	Post-surgical analysis	No	Yes	32
Jiang	2024	Asia	F	R	1,17	AML	Post-surgical analysis	No	Yes	45
Jiang	2024	Asia	F	L	1,25	AML	Post-surgical analysis	No	Yes	26
Jiang	2024	Asia	M	L	10,18	AML	Post-surgical analysis	No	Yes	19
Jiang	2024	Asia	M	R	1,14	AML	Post-surgical analysis	No	Yes	11
Jiang	2024	Asia	F	R	2,67	AML	Post-surgical analysis	No	Yes	14
Jiang	2024	Asia	F	R	16,5	AML	Post-surgical analysis	No	Yes	7
Jiang	2024	Asia	F	L	6	AML	Post-surgical analysis	No	Yes	128
Jiang	2024	Asia	F	R	4,4	AML	Post-surgical analysis	No	Yes	102
Jiang	2024	Asia	F	L	3,86	AML	Post-surgical analysis	No	Yes	180
Jiang	2024	Asia	M	L	3,6	AML	Post-surgical analysis	No	Yes	59
Jiang	2024	Asia	F	R	4,65	AML	Post-surgical analysis	No	Yes	28
Jiang	2024	Asia	F	R	7,5	AML	Post-surgical analysis	No	Yes	49
Jiang	2024	Asia	F	L	3,6	AML	Post-surgical analysis	No	Yes	49
Jiang	2024	Asia	F	L	11,15	AML	Post-surgical analysis	No	Yes	240
Jiang	2024	Asia	F	R	4,25	AML	Post-surgical analysis	No	Yes	96
Kvietkauskas	2024	Europe	F	R	.	PEComa-NOS	Post-surgical analysis	No	Yes	40
Lee	2024	Asia	F	R	5,5	PEComa-NOS	Post-surgical analysis	No	Yes	12
Liu	2024	Asia	F	R	1,6	PEComa-NOS	Post-surgical analysis	No	Yes	36

Tababi	2024	Africa	F	R	1,7	AML	Biopsy	No	Yes	15
Takada	2024	Asia	F	R	.	PEComa-NOS	Post-surgical analysis	No	Yes	16
Tan	2024	Asia	F	C	11	AML	Post-surgical analysis	No	Yes	6
Vijayanirmala	2024	Asia	F	R	10	PEComa-NOS	Post-surgical analysis	No	Yes	36
Vijayanirmala	2024	Asia	F	R	6	PEComa-NOS	Post-surgical analysis	No	Yes	11
Vijayanirmala	2024	Asia	F	R	2,8	PEComa-NOS	Post-surgical analysis	No	Yes	8
Yang	2024	Asia	M	R	24	PEComa-NOS	Radiology	Yes	Yes	18
Yang	2024	Asia	F	R	2,2	AML	Post-surgical analysis	No	Yes	84
Yazici	2024	Asia	F	R	3,5	clear cell sugar tumor	Post-surgical analysis	No	Yes	66
Zaidi	2024	Asia	F	R,L,C	10	CCMMT of falciform ligament	Post-surgical analysis	No	Yes	6
Zhu	2024	Asia	F	R	3,5	PEComa-NOS	Post-surgical analysis	No	Yes	12
Abe	2025	Asia	F	L	0,8	PEComa-NOS	Biopsy	No	No	12
Boccatonda	2025	Europe	F	L	3,8	PEComa-NOS	Biopsy	No	Yes	48
Guo	2025	Asia	M	L	6,5	PEComa-NOS	Post-surgical analysis	No	Yes	12
Paula	2025	Europe	F	R	4,7	PEComa-NOS	Post-surgical analysis	No	Yes	36
Saadoun	2025	Europe	M	L	11	PEComa-NOS	Biopsy	No	Yes	24

Abbreviations: PEComa, Primary Hepatic Perivascular Epithelioid Cell Tumor; FU, follow up; F, female; M, male; R, right; L, left; C, caudate; AML, angiomyolipoma; PEComa-NOS, PEComa not otherwise specified; CCMMT, clear cell myomelanocytic tumor.
